# Evidence of Exacerbated Gender Inequality in Child Care Obligations in Canada and Australia during the COVID-19 Pandemic

**DOI:** 10.1017/S1743923X20000574

**Published:** 2020-12

**Authors:** Regan M. Johnston, Anwar Mohammed, Clifton van der Linden

**Affiliations:** 1 McMaster University; 2 McMaster University; 3 McMaster University

**Keywords:** COVID-19, gender, child care, Canada, Australia

## Abstract

Households in Canada and Australia have exhibited similar trends in the gendered allocation of additional child care responsibilities resulting from policy responses to the COVID-19 pandemic. In this article, we employ survey data to analyze the extent to which policy interventions related to COVID-19 have exacerbated gender disparities in child care obligations. We find that existing asymmetrical distributions of child care obligations in Canada and Australia have been amplified during the pandemic, resulting in a disproportionate burden on women. During the pandemic we also find that, in households with children, women tend to report experiencing poorer mental health than men.

Pandemics have historically exacerbated gender inequalities (Wenham, Smith, and Morgan 2020), and coronavirus disease 2019 (COVID-19) has been no exception. COVID-19 has been linked to increased gender-based violence (Taub 2020), disproportionate loss of employment among women (Alon et al. 2020), and an increased demand for unpaid care work (United Nations 2020). In this article, we demonstrate how certain policy interventions intended to prevent the spread of COVID-19 have compounded existing gender disparities around child care obligations. In particular, we examine the gendered implications of stay-at-home measures in Canada and Australia.

We employed survey data in Canadian and Australian households with children under the age of 15 to compare the average differences between men and women in terms of hours per week spent on child care prior to and during the pandemic. We found that in Canada, the average woman with children at home reported spending nearly 50 more hours per week on child care during the pandemic than the average man. Although both women and men, on average, reported a 39% increase in the number of hours per week spent on child care during the pandemic, women were already spending more than double the number of hours per week on child care than men prior to the pandemic. Similarly, women in Australian households with children reported spending approximately 43 more hours per week on child care during the pandemic than men. We also found that among households with children, women in both Canada and Australia reported lower average mental health scores than did men, although only in the Canadian case were these results statistically significant.

Our analysis demonstrates how certain policy interventions related to COVID-19 exacerbated the preexisting asymmetrical division of household labor between men and women (Dinh, Strazdins, and Welsh 2017; Orloff 2002). This research contributes to a growing body of evidence on the gendered implications of policy measures related to COVID-19 (Funk 2020; Shay 2020). In particular, we provide empirical validation of claims that gender disparities in households with children have been amplified as a result of the pandemic. We also indicate the potential gendered impact of COVID-19 and associated policy interventions on the mental health of women with children at home. Our findings support assertions that women with child care obligations have seen a dramatic and disproportionate increase in invisible labor as a result of the COVID-19 pandemic.

## GENDER AND CARING WORK

By most accounts, women's increasing labor force participation has not produced a more balanced gendered division of domestic labor (Blossfeld and Drobnič 2001; Gershuny 2000; Hochschild 1989). Women are likely to spend more time on child care regardless of hours spent in paid employment (Craig 2006; Presland and Antill 1987).

Despite continued gendered disparities in average employment earnings, the persistent asymmetric allocation of caring work between women and men is not solely a reflection of relative differences in income. Rather, it belies social constructions of gender roles that position the primary role of women as caregivers and nurturers (Chick, Heilman-Houser, and Hunter 2002; West and Zimmerman 1987). These roles are so culturally entrenched that even “as wives become the primary breadwinners, they do more of the domestic tasks to reinforce traditional gender identities” (Breen and Cooke 2005, 43).

## COVID-19 POLICY RESPONSES IN CANADA AND AUSTRALIA

Canada and Australia serve as useful case comparisons given their structural and institutional similarities. Both have federal governments that use the Westminster parliamentary system. Moreover, despite certain noteworthy exceptions, both countries have similar views in terms of gender equality (Horowitz and Fetterolf 2020). Canada and Australia are among the highest-ranking countries in the G-20 in terms of civil service gender equality initiatives (Global Government Forum 2020), have similar gender balances in terms of enrolment in tertiary education (Conference Board of Canada 2011), and have a roughly equivalent gender pay gap (Chamberlain, Zhao, and Stansell 2019). This is by no means an exhaustive manifest of the available comparisons between the two countries, but they are sufficiently similar to expect comparable gendered implications as a result of a worldwide pandemic such as COVID-19.

A confounding factor in the analysis of said implications, however, is variation in policy responses to COVID-19 between the two countries. In Canada, most provincial authorities closed schools and child care centers as of mid-March (Young 2020). This measure, combined with other restrictions implemented in most provinces that effectively restricted households from drawing on extended family or other child care supports, left most parents or guardians with little other choice than to assume full-time child care responsibilities. Despite stay-at-home measures commencing at approximately the same time as in Canada, the Australian government was reticent to close schools, although numerous state authorities either brought school holidays forward or explicitly asked parents to keep children at home (Karp 2020; Ting and Palmer 2020). To support preschool-aged children, the federal government introduced special measures to support child care across the country, including making it free to working parents from April 2 until July 13, 2020 (Australian Bureau of Statistics 2020).

## DATA AND METHOD

To analyze the gendered differences in child care responsibilities prior to and during the pandemic, we employ survey data from the COVID-19 Monitor, which is an ongoing public opinion study conducted by research firm Vox Pop Labs (see Mohammed, Johnston, and van der Linden 2020; van der Linden and Savoie 2020). The study involves weekly survey waves fielded in Canada beginning March 20, 2020, and in Australia beginning April 17, 2020. Each sample was drawn from the Vox Pop Labs online respondent panel, which consists of approximately 650,000 Canadian and 600,000 Australian panelists. The sample selected for each wave of the Canadian and Australian studies was prestratified on the basis of sex, age, education, region, and partisan affiliation so as to approximate a nationally representative sample.

In two separate waves of the study fielded in Canada and Australia from April 24 to 28, 2020, and June 1 to 5, 2020, respondents were asked the following question: “Prior to the COVID-19 pandemic, how many hours per week on average did you spend on each of the following?” A number of activities were listed, including “Looking after one or more children living inside your household, without pay.” For each activity, respondents were asked to indicate a number of hours spent by using a numeric entry text box. Respondents were then immediately asked the following question: “How many hours per week on average did you spend on each of the following last week?” The same list of activities was presented to respondents, and again, they were asked to indicate the number of hours spent on each activity in the last week. The combined sample size for the Canadian waves that contained this battery of questions was 4,070 and that of the Australian waves was 3,676.

In each wave of both the Canadian and Australian studies, respondents were asked, “At present, how would you characterize your mental health?” The response scale items included the following: “poor,” “fair,” “good,” “very good,” and “excellent.”

Responses to these survey items reported here have poststratified by modeling raking weights for each wave independently using marginal distributions from the 2016 Canadian census and the 2016 Australian census, respectively, as well as from the 2019 Canadian and Australian federal elections. Raking weights were modeled based on the joint distributions of self-reported sex, age, education, region, and partisanship of each observation and the corresponding marginal distributions in the relevant census and electoral registry. Weighted averages were then generated for each of the variables of interest presented in the following results. This procedure permits us to confidently make representative inferences about the Canadian and Australian populations and subpopulations of interest.

## RESULTS

### Time Spent on Child Care

**Figure 1.**
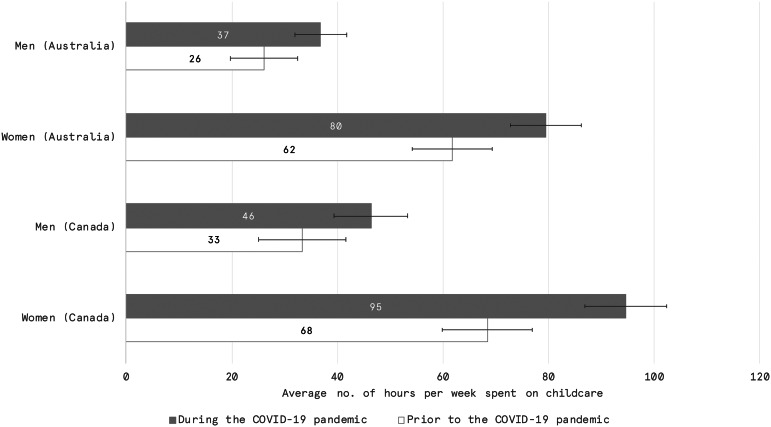
Average number of hours per week spent looking after one or more children living inside one's household, without pay. Error bars represent 95% confidence intervals which were calculated using the survey package in R and consider the weighting structure. *Source:* Vox Pop Labs, COVID-19 Monitor (*n*_Australia_ = 3,676; *n*_Canada_ = 4,070).

Figure 1 summarizes the results of self-reported hours spent on child care both prior to and during the pandemic. The sample is subset by respondents who indicate that they live in a household with one or more children under the age of 15. Differences between genders and between time periods are significant at a 95% confidence interval for the mean values associated with the number of hours spent looking after one or more children living inside one's household.

Among Canadian men with children in their household, we observe an increase in the average number of hours per week spent on child care from 33 hours prior to the pandemic to 46 hours during the pandemic. Note that this difference is not statistically significant. Among Canadian women that figure rises from 68 hours per week prior to the pandemic to 95 hours during the pandemic. While both Canadian men and women report an equivalent relative increase in the number of additional hours spent on child care obligations (37%), in absolute terms women are still allocating 2.5 times more hours per week to child care obligations than men. The increased demand on parents or guardians to provide child care during the pandemic has multiplied the disparity already prevalent in Canadian households prior to the pandemic.

Similar findings are observable in the Australian data. Prior to the pandemic, the average Australian man in a household with children under 15 years of age reported spending 26 hours a week on child care. By comparison, the average Australian woman reported spending 62 hours a week caring for children in the household. During the pandemic, those figures increase to 37 hours for men and 80 hours for women. This represents a 42% increase for men (although, again, the difference for men is not statistically significant) and a 29% increase for women. Women are yet again allocating more than twice as many hours as men to child care responsibilities during the pandemic.

The difference between the average number of hours per week allocated to child care responsibilities in Canada versus Australia is arguably related to differing policy measures in terms of child care supports during the pandemic. Though there is also a difference between the two countries in time spent on child care prior to the pandemic, with Australian parents and guardians reporting slightly lower averages than their Canadian counterparts, these differences are not statistically significant. However, there is a statistically significant difference in time spent on child care between women in Canada and Australia during the pandemic. Child care centers in Canada were closed during the height of stay-at-home orders in most provinces, but in Australia they not only remained open but were fully subsidized by the federal government for working parents. This may explain at least part of the difference.

### Mental Health

**Figure 2.**
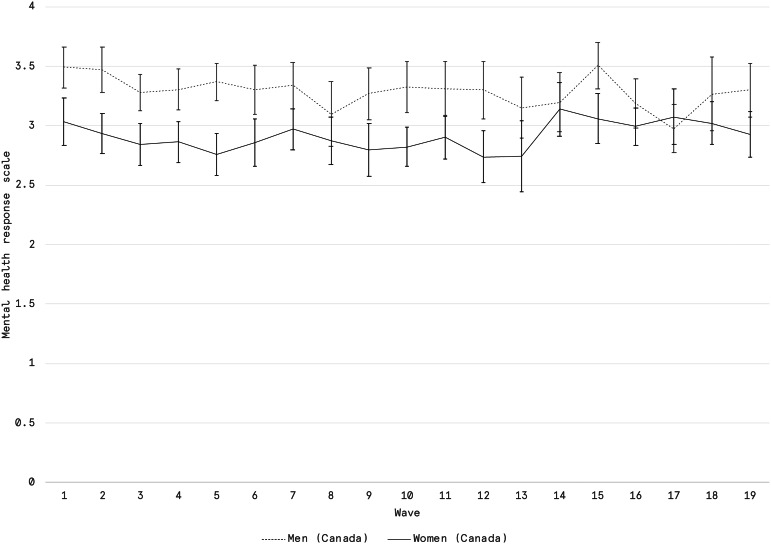
Average self-reported mental health among men and women in Canadian households with children under the age of 15. Scale is an ordinal Likert scale from 0 to 4, with associated scale items being “poor,” “fair,” “good,” “very good,” “excellent”. Error bars represent 95% confidence intervals which were calculated using the survey package in R and consider the weighting structure. *Source:* Vox Pop Labs, COVID-19 Monitor (*n* = 41,025).

In our examination of the gendered dimensions of mental health in reaction to the COVID-19 pandemic, we observe that Canadian women in households with children under 15 report worse overall mental health outcomes than men. Figure 2 illustrates a time series running from mid-March to late July 2020 of self-reported mental health scores on a 5-point Likert scale. The average weekly score for women in the time series consistently falls below that of men. This pattern is statistically significant at a 95% confidence level up until Wave 13, which ran from June 5 to 9. The overall mean for across the time series is 2.9 for women and 3.3 for men. A value of 3 on the associated Likert scale corresponds with a “very good” rating on mental health, and the mean values for both men and women in households with children hover around this mark. Although the difference between men and women is substantively negligible, it is statistically significant.

**Figure 3.**
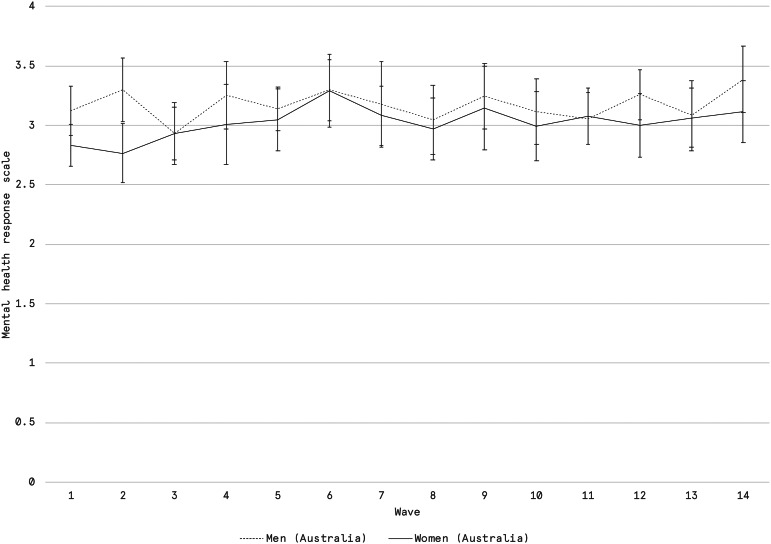
Average self-reported mental health among men and women in Australian households with children under the age of 15. Scale is an ordinal Likert scale from 0 to 4, with associated scale items being “poor,” “fair,” “good,” “very good,” “excellent”. Error bars represent 95% confidence intervals which were calculated using the survey package in R and consider the weighting structure. *Source:* Vox Pop Labs. COVID-19 Monitor (*n* = 27,459).

In Australia, there is no discernible difference in self-reported mental health between women and men in households with children. In Figure 3 we plot the time series of average mental health scores among Australians from mid-April onwards. Women consistently demonstrate a lower average rating but, aside from Wave 2, which ran from April 24 to 28, the differences between men and women in Australia are not statistically significant.

The findings suggest that despite evidence of a disproportionate allocation of additional child care responsibilities falling to mothers and female guardians during the pandemic, women exhibited high levels of emotional resilience. We do not have a baseline from before the pandemic for comparison, so it is unclear whether mental health declined among women (or men) at the outset of the pandemic. However, averaged ratings of mental health remain relatively consistent from mid-March 2020 onwards. Only among Canadian women do we find a significant, if not substantive, difference in self-reported mental health throughout the pandemic. Again, this may be in part explained by the lack of access to child care supports that were made available to Australian households, and could very reasonably reflect the extraordinary and disproportionate additional child care responsibilities that Canadian women in particular ultimately shouldered.

## DISCUSSION

The findings presented in this research note demonstrate that existing asymmetrical distributions of invisible labor—child care in particular—have been substantially exacerbated as a result of restrictive policies imposed in response to the COVID-19 pandemic. In both Canada and Australia, women in households with children under the age of 15 reported spending twice as many additional hours per week on child care as a result of the pandemic. Given that, on average, women in both countries were already devoting more than twice as many hours as men to child care responsibilities *prior to* the pandemic, COVID-19 has only further exacerbated existing gender inequalities in both countries in the context of child care.

In absolute terms, the mean Australian woman in a household with children has spent an additional 18 hours per week on child care duties during the pandemic. Likewise, the mean Canadian woman in a household with children reports allocating an additional 27 hours per week to child care, resulting in an average of 95 hours per week—or nearly 14 hours per day—taking care of children during the pandemic. Even though our sample includes both stay-at-home parents and those employed outside the home, the deleterious career effects for women in the latter category seem almost unavoidable.

These findings demonstrate the need for gender-targeted policy measures to address the setbacks to gender equality that have resulted from earlier policy interventions related to COVID-19. Such measures should both address the present disparities between men and women within Canadian and Australian households but must also factor into their design the lasting implications COVID-19 will likely have on the pursuit of gender equality.

Regan M. Johnston is a PhD student in the Department of Political Science at McMaster University and a doctoral fellow with the Digital Society Lab: johnsr27@mcmaster.ca; Anwar Mohammed is a PhD student in the Department of Political Science at McMaster University and a doctoral fellow with the Digital Society Lab; mohaa33@mcmaster.ca; Clifton van der Linden is Assistant Professor in the Department of Political Science and director of the Digital Society Lab at McMaster University. He is also the founder and chief executive officer of Vox Pop Labs: cliff.vanderlinden@mcmaster.ca
